# The Sanicula Marilandica a Cure for the Bite of a Snake

**Published:** 1842-05

**Authors:** 


### THE SANICULA MARILANDICA A CURE FOR THE BITE OF A SNAKE.

Some years since we received from Mr. J. F. Hamtramck of Missouri, an imperfect specimen of a plant said to be an efficacious remedy for snake-bite. On submitting it, a short time since, to Professor Short, whose knowledge of the botany of the central parts of the United States is most full and accurate, he decided that it was the *Sanicula Marilandica.* The following is a copy of the note which accompanied the specimen:—

"The root is the part employed as containing the medicinal qualities, and is used in three different ways; most generally the Indians carry the root about them in its natural dried state, and to apply it, masticate it sufficiently to form a full cataplasm, which they bind immediately over the wound. Others, again, pulverize the dried root, and carry it about them in a leathern pouch; when it is to be applied, it is previously moistened. Others, lastly, use it in its green state, in which case it is bruised into a mass. They always scarify the wound previous to the application. During a hunting tour through the upper part of Texas, which I took with the Osage nation in the fall of 1828, an Indian girl was bitten, as I was told, about 12 o'clock, while searching along a small stream for fire wood. We were encamped, and all the Indians out on the hunt. About an hour before sunset I got into camp, and was informed of the misfortune of the poor girl. On visiting her, I found her leg swollen to more than twice its usual size (she had been bitten on the outside of the leg, about midway between the knee and ankle), and she was apparently suffering the most excruciating agony. On the arrival of the first person from the hunt who had any of the root with him, we set about masticating it, while an old woman scarified the wound with the point of a butcher-knife, which the poor girl seemed not to feel in the least. A good large poultice was applied about dark; on the next morning, the swelling had entirely subsided, and the girl travelled on foot, from before day light, till at least 12 o'clock." D.

